# Engineering a Novel Self-Assembled Multi-siRNA Nanocaged Architecture with Controlled Enzyme-Mediated siRNA Release

**DOI:** 10.1021/acsami.2c15086

**Published:** 2022-12-15

**Authors:** Pedro M. D. Moreno, João Cortinhas, Ana S. Martins, Ana P. Pêgo

**Affiliations:** † i3S - Instituto de Investigação e Inovação em Saúde, Universidade do Porto, 4200-135 Porto, Portugal; ‡ INEB - Instituto de Engenharia Biomédica, Universidade do Porto, 4200-135 Porto, Portugal; § Faculdade de Engenharia da Universidade do Porto, 4200-465 Porto, Portugal; ∥ Instituto de Ciências Biomédicas Abel Salazar (ICBAS), Universidade do Porto, 4050-313 Porto, Portugal

**Keywords:** siRNA, nanostructure, self-assembly, gene silencing, conjugates

## Abstract

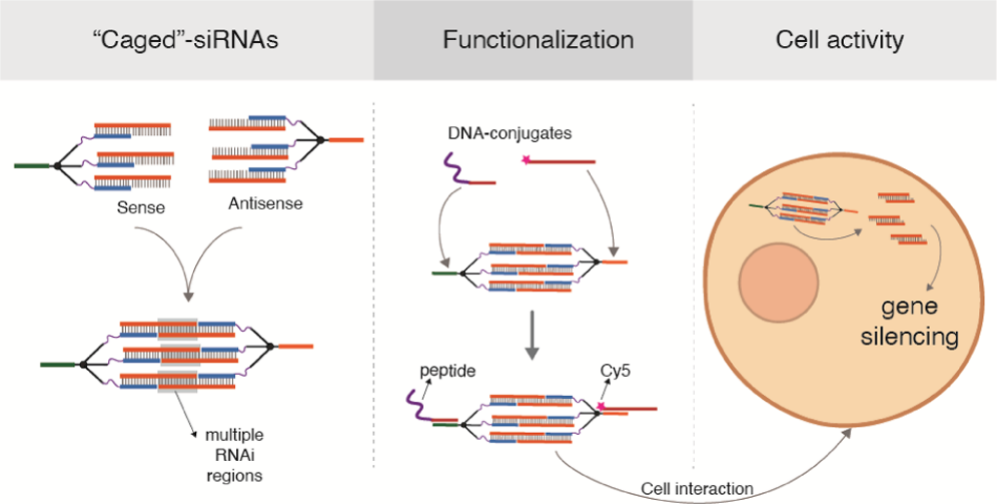

The RNA interference (RNAi) chemical and structural design space has evolved since its original definitions. Although this has led to the development of RNAi molecules that are starting to address the issues of silencing efficiency and delivery to target organs and cells, there is an on-going interest to improve upon their properties to attain wider therapeutic applicability. Taking advantage of the flexibility given by DNA and RNA structural and chemical properties, we here investigated unconventional RNAi encoding structures, designated by caged-siRNA structures (CsiRNAs), to explore novel features that could translate into advantageous properties for cellular delivery and intracellular activity. Using the principles of controlled nucleic acid self-assembly, branched DNA–RNA hybrid intermediates were formed, ultimately leading to the assembly of a “closed” structure encompassing multiple RNAi units. The RNAi active regions are further triggered by an encoded RNAse H-mediated release mechanism, while the overall structure possesses easily addressable anchors for hybridization-based functionalization with active biological moieties. We confirmed the production of correct structures and demonstrated that the encoded RNAi sequences maintain gene silencing activity even within this novel unconventional nanoarchitecture, aided by the intracellularly triggered RNAse H release mechanism. With this design, functionalization is easily achieved with no negative effects on the silencing activity, warranting further development of these novel molecular structures as a multi-RNAi platform for therapeutic delivery.

## Introduction

The promise of small interfering RNA (siRNA) as a potent gene drug in medicine has sustained its continued development, pursuing, among others, improved and specific cellular uptake, reduced sensitivity to degradation, prolonged silencing action, as well as reduction of off-target and immune activation effects. Apart from different delivery options under development, also, modifications to the siRNA molecule have been proposed to deal with some of these challenges. These can not only include the use of chemically modified nucleosides and phosphodiester backbones but also changes to the structure of the double-stranded RNA or overall RNA architecture.^1,2^ Some of the more prominent structural modifications to synthetic siRNAs that have been explored use simple designs such as the use of extended sense and antisense strands (25/27 nt) to form Dicer substrate siRNAs (DsiRNA)^3^ or the small internally segmented interfering RNA (sisiRNA) formed by two shorter sense strands hybridized to a regular intact antisense strand.^4^ An effort to increase stability of siRNA molecules led to the development of synthetic double-stranded siRNA using a circular single RNA chain in a symmetrical double hairpin configuration, the designated dumbbell siRNA.^5^ Further increases in complexity have been obtained by principles of branching, either through synthesis or self-assembly processes forming interesting nanostructured siRNAs. These include the two- or four-arm branched siRNA, connected by symmetric doubler phosphoramidites,^6^ or the designated tripodal interfering RNAs, where siRNA units are connected through the use of a trebler phosphoramidite core, which was extended with DNA linkers.^7^ A variation on the branched siRNA structures was also introduced by the design of extended RNAs forming three- or four-way junctions through self-assembly^8^ and by the use of the phi29 DNA-packaging RNA (pRNA) as a scaffold to which siRNAs append and create branched structures.^9^

With the approval, at the moment of writing, of five siRNA drugs (patisiran; givosiran; lumasiran; inclisiran; vutrisiran), the RNAi therapeutics field is now delivering on its promise. This has led to a renewed and enhanced interest in developing novel RNAi trigger designs that can achieve robust and safe RNAi in locations other than the liver and that may be directed toward not only disorders of a single causative gene but also to more common and complex disorders. Toward that goal, branched RNA structures, achieved through synthesis using branching amidites or self-assembly, have the potential to carry multiple siRNAs in a single molecularly defined unit enabling uptake of multiple siRNAs per cell uptake event. Also, depending on the design, the silencing of multiple genes is possible to achieve. Overall, the principles of branching can be used toward the design of increasingly complex RNA nanostructures with the potential to act as programmable carriers of different bioactive nucleic acid therapeutics.^10^

Here, we have combined dendritic-like DNA building blocks, achieved by synthesis-based branching, with nucleic acid self-assembly to explore a new RNAi nanoarchitecture. In this self-assembled architecture, multiple functional RNAi triggers are locked inside a bimolecular structure resembling a closed cage- or cryptand-type structure, as previously designated for DNA dendrimer-based bimolecular structures.^11^ It is also molecularly analogous to the acetylene bond, thus also referred to as a nanoacetylene structure type.^12,13^ We explore this novel RNAi nanoarchitecture, evaluating the assembly as well as mechanisms of release of the RNAi triggers and intracellular gene silencing capacity. These caged-RNAi structures could present novel opportunities for cell delivery of multiple siRNA units or as part of more complex nucleic acid supramolecular structures with multiple biological activities.

## Materials and Methods

### Oligonucleotides

All oligonucleotides used were purchased from Integrated DNA Technologies, except the trebler sequences (Oligonucleotide Synthesis Facility, Yale University) and all were subjected to HPLC purification.

Sequences are presented in Table 1.

**Table 1:** Oligonucleotide Sequences Utilized^a^

name	sequence (5′-3′)
trebler A_S	TGTGCTTGTGATTGATGT-(spacer 18)-(trebler)-CAATAATGACTAAAAGCG
trebler A_AS	CTTGTCTCGTTTCTATCT-(spacer 18)-(trebler)-AAGACTCAGGAAAAGCGA
trebler B_S	CGCGCCGACATCCAGTCG-(spacer 18)-(trebler)-CAATAATGACTAAAAGCGACG
trebler B_AS	CGCGGCGCCGATACGACG-(spacer 18)-(trebler)-GGCAACCAATATACAATGGCG
sense D–R strand (GFP)	ACATCAATCACAAGCACArUrGrArCrCrCrUrGrArArGrUrUrCrArUrCrUrGrCrArCrCrArCrCrG
antisense D–R strand (GFP)	AGATAGAAACGAGACAAGrCrGrGrUrGrGrUrGmCrAmGrAmUrGmArAmCrUmUrCrArGrGrGrUrCrA
sense D–R strand A (PTEN)	ACATCAATCACAAGCACArUrUrCrGrArCrUrUrArGrArCrUrUrGrArCrCrUrArUrArUrUrUrArU
antisense D–R strand A (PTEN)	AGATAGAAACGAGACAAGrArUrArArArUrArUmArGmGrUmCrAmArGmUrCmUrArArGrUrCrGrArA
sense D–R strand version I (GFP)	CGACrUrGrGrArUrGrUrCrGrGCGCGmAmCmCrCrUmGrAmArGmUrUrCrArUrCrUrGrCrA
antisense D–R strand version I (GFP)	CGTCrGrUrArUrCrGrGrCrGrCCGCGrUrGrCrArGrArUrGmArAmCrUmUrCmArGmGrGmUTT
sense D–R strand version II (GFP)	CGACTGGArUrGrUrCrGrGrCrGrCrGmAmCmCrCrUmGrAmArGmUrUrCrArUrCrUrGrCrA
antisense D–R strand version II (GFP)	CGTCGTATrCrGrGrCrGrCrCrGrCrGrUrGrCrArGrArUrGmArAmCrUmUrCmArGmGrGmUTT
DNA sense arm	CGCGCCGACATCCAGTCG
DNA antisense arm	CGCGGCGCCGATACGACG
siGFP	AS: 5′-UGmCAmGAmUGmAAmCUmUCmAGmGGmUCmA; S: 3′-CCACGUCUACUUGAAGUmCmCmCA
Tet1-DNA conjugate	HLNILSTLWKYRC-(spacer 18)-CGTCGCTTTTAGTCATTATTG
_Cy5_PStail	T*T*T* T*T*T* CGC CAT TGT ATA TTG GTT GCC-(spacer 9)-Cy5

a Notes: green fluorescence protein (GFP); phosphatase and tensin homologue (PTEN). Concentrations were estimated by measuring absorbance at 260 nm using a NanoDrop 1000 Spectrophotometer (Thermo Fisher Scientific). DNA = N, RNA = rN, 2′-*O*-methyl RNA = mN, 2′ fluoro = fN. Spacer 18 = hexaethylene glycol; spacer 9 = triethylene glycol spacer; trebler = long trebler branching unit (4-armed) (see Figure 1A for details).

### Oligonucleotide Structure Assembly, Purification, and Characterization

#### Double-Stranded Oligonucleotide Structures

Where only double-stranded linear oligonucleotide sequences were used (not involving the assembly with treblers), annealing was performed in 30 mM Tris-HCl, 50 mM NaCl, pH 7.3 buffer in a thermocycler with the following temperature program: 95 °C, 2 min → slow cool for 30 min to 20 °C → 20 °C, 5 min → 4 °C. Afterward, if blocking DNA arms were used, these were annealed in a second step by incubating for 1 h at room temperature in a 1:1 ratio.

#### Caged-siRNAs Assembly

Caged-siRNAs were assembled in a two-step reaction using the following assembly buffer: 30 mM Tris-HCl, 50 mM NaCl, pH 7.3. A typical assembly consisted in a first annealing reaction of the treblers S and AS with the respective and complementary RNA sense and antisense strands made in a thermocycler with the following temperature program: 85 °C, 5 min → 50 °C, 60 min → 4 °C. After the first annealing, a dendrimeric-like structure was formed and designated as Branch S (carrying three sense RNA sequences while leaving one free ssDNA anchor sequence) or Branch AS (carrying three antisense RNA sequences, while leaving one free ssDNA anchor sequence). A second annealing reaction was then followed by mixing both Branch structures at a theoretical ratio of 1:1 to achieve the final caged-siRNA structure. Again, this was achieved in a thermocycler using the following temperature program: 50 °C, 45 min → 20 °C, 5 min → 4 °C. For some sequences, temperature programs had minor adjustments indicated in the respective figures.

All oligonucleotide assemblies were typically characterized by PAGE (polyacrylamide gel electrophoresis) using 30% acrylamide/bis (29:1 ratio) solution (Bio-Rad), 10× TBE (tris borate EDTA) buffer (NZYTech). Polyacrylamide gels typically consisted in a top 4% stacking layer and a 6% resolving layer and were run using a Mini-PROTEAN Tetra Cell System (Bio-Rad). All gels were stained with SYBRGold (Thermo Fisher Scientific) for 8–10 min and were imaged in a GelDoc XR^+^ Imaging System (Bio-Rad). Analysis of gels and quantification of bands for calculation of assembly yields were done using volume analysis tools for quantification of the adjusted band volume intensity in relation to the whole lane with global background subtraction, using the Image Lab 6.0 software (Bio-Rad).

#### Gel Purification

For caged-siRNA purification, the structures were first run through a preparatory 4–6% polyacrylamide gel for 100 min at 80 V. Later, the gel was visualized through the UV shadow technique using a fluorescent TLC silica gel 60 F_254_ plate (Sigma-Aldrich) and a UV light source. The corresponding caged-siRNA bands were cut with a scalpel. The gel slices were then transferred to a 0.6 mL microcentrifuge tube (Axygen, Maximum Recovery) with a hole in the bottom, made with a 19G needle, and inserted in a bigger 1.5 mL microcentrifuge tube (Axygen, Maxymum Recovery). Centrifugation was then performed at room temperature for 8 min at 6000*g*. The resulting gel slurries were transferred to 0.22 μm Corning Costar Spin-X columns (Sigma-Aldrich) with 500 μL of assembly buffer and were subjected to three incubations (overnight + 6 h + overnight) at 15 °C, with agitation at 1400 rpm in a ThermoMixer (Eppendorf). After each incubation, the columns were centrifuged for 5 min at 13 000*g* and the supernatant collected. In the end, all of the supernatants were joined and concentrated through an Amicon 3 kDa column (Merck), according to manufacturer’s instructions. Alternatively, for the caged-siRNA structures using the heavily modified RNA S (sense) and AS (antisense) strands, the purification followed an agarose gel electroelution protocol adapted from ref (14). Briefly, the RNA structures were run in a 2.5% (w/v) resolving agarose layer on top of a preformed thin 4% (w/v) agarose layer (made in 1× TBE). The agarose gel was run inside an ice bath at 80 V. One designated marker lane was used where a small amount of 10 pmol of the caged-siRNA structures was loaded to allow visualization of the run. For that, the gel area corresponding to the marker lane was cut out to allow staining with SYBRGold (1:5000, 30 min). The portion of the gel containing the samples to be purified was left unstained. After the separation phase, a well was cut in front of the band corresponding to the correct structures. The well was then filled with a solution composed of 30% sucrose in 1× TBE. The gel run was then resumed for the band to accumulate in the well containing the sucrose solution. This solution containing the caged-siRNA was then pipetted from the well and loaded in an Amicon 3 kDa column (Merck) for a buffer exchange to the original assembly buffer (as per manufacturer’s instructions). The purified structure was confirmed by PAGE analysis as described above.

#### TEM Characterization

For negative-staining transmission electron microscopy (TEM), Formvar/carbon film-coated mesh nickel grids (Electron Microscopy Sciences, Hatfield, PA) were first glow-discharged for 15 s. Next, the grids were treated with 5 μL of a 1 μg/mL poly(d-lysine) (PDL) (#P0296, Merck) aqueous solution for 30 s, followed by draining with filter paper (Whatman). A volume of 5 μL of purified anti-GFP cage structures (0.1 μM) was added on top of the grids and left standing for 2 min. The liquid in excess was removed with filter paper, and 5 μL of 2% (w/v) uranyl acetate was added onto the grids and left standing for 10 s, after which the liquid in excess was removed with filter paper. The grids were then washed three times by placing them inverted on top of nuclease-free water (Qiagen) drops, formed on parafilm, for 5 s, without removing the excess liquid in between washes. Visualization was carried out on a JEOL JEM 1400 TEM at 80 kV (Tokyo, Japan). Images were digitally recorded using a CCD digital camera Orious 1100 W Tokyo, Japan. Micrographs were processed with the Scipion software package^15^ using the Xmipp3 plugin^16^ for supervised semiautomatic particle picking. In total, 496 particles were then selected manually and measured with the help of Image J software (version 2.0.0-rc-69/1.52p).

#### In Vitro Digestions with Dicer and RNAse H Enzymes

For *in vitro* Dicer digestion assays, Genlantis Recombinant Human Dicer Enzyme Kit was used. Samples were incubated according to kit instructions, with 2 units of recombinant Dicer in a volume of 15 μL at 37 °C for 12 h. Later, 2 μL of stop solution was added.

For RNAse H cleavage assays, samples were incubated with RNAse H enzyme (0.5 U/μL, New England BioLabs) in a modified incubation buffer consisting of 31.5 mM Tris-HCl, 50 mM NaCl, 3 mM Mg, pH 7.3, in a final volume of 10 μL for 15 min at 37 °C.

Samples from the Dicer and RNAse H digestion assay were loaded and run on a native PAGE and stained with SYBRGold (Thermo Fisher) for visualization on a GelDoc imaging system (Bio-Rad) as described above.

### In Vitro Cell Culture Assays

#### Cellular Transfections

U2OS-GFPLuc cells (based on developments made in ref (17) and kindly supplied by the lab of Dr. Edvard Smith, Karolinska Institute) constitutively expressing the fusion gene GFP-Luciferase were cultured in Dulbecco’s Modified Eagle Medium (DMEM) with GlutaMAX (Gibco), supplemented with 10% (v/v) fetal bovine serum (FBS, Gibco), heat-inactivated, and 0.1% gentamycin (v/v) (Gibco). Cells were passaged every 2–3 days at a ratio of 1:10/15.

For U2OS-GFPLuc transfections, cells were plated in 96-well tissue culture plates (Corning) at a density of 8000 (for the transfections with CsiRNAs) or 9000 (assessed by the Trypan blue assay) viable cells (for the transfections with oligos A–D) per well and transfected 24 h later using Lipofectamine RNAiMax (Thermo Fisher Scientific). Transfections followed manufacturer’s instructions with a ratio of 0.25 μL of RNAiMAX per 10 μL of volume. For transfections with CsiRNAs with time points of 96 h, cells were trypsinized after 72 h and replated in a 48-well tissue culture plate.

For analysis of GFP-Luciferase silencing, cells were washed two times with phosphate buffer saline (PBS) 1×. Afterward, cells were lysed in a lysis buffer (PBS 1×, 0.15% (v/v) Triton X-100) for 30 min at 4 °C. A volume of 20 μL of lysate and 100 μL of Luciferase Assay Reagent (Promega) were added per well to a 96-well flat bottom white plate (Nunc), and luminescence was immediately read in a SynergyMx MultiMode Microplate Reader (BioTek). Luminescence results were normalized with the use of Micro BCA Protein Assay Kit (Thermo Fisher Scientific). In brief, 50 μL of lysate and 100 μL of Micro BCA Working Reagent were added per well to a 96-well plate. The plate was incubated at 37 °C and after 2 h, absorbance was read at 562 nm in a SynergyMx MultiMode Microplate Reader. A standard curve with the provided assay kit bovine serum albumin was used to determine the protein concentration in each sample.

#### Cell Uptake Assays

ND7/23 neuroblastoma cells were cultured in DMEM GlutaMAX (Gibco), supplemented with 10% (v/v) FBS (Gibco), heat-inactivated, and 0.1% gentamycin (v/v). Cells were passaged every 2–3 days at a ratio of 1:10–15.

ND7/23 cells were plated in μ-Slide 18 well IbiTreat (IBIDI) slides at a density of 10 000 viable cells per well. After 48 h, the cell culture medium was replaced by Opti-MEM (Gibco) containing H-CsiRNAs in solution and the plate was incubated for 4 h at 37 °C. Cells were then subjected to a two-step fixation protocol involving partial removal of 75% of the cell medium volume and adding PBS up to maximum well volume (3×) and on the last partial wash adding equal volume of 4% paraformaldehyde solution in 1× PBS. Cells were then left to fix for 10 min at RT. Then, the cells were washed twice with 1× PBS and a new solution of 3% paraformaldehyde in PBS was added to the cells and left to incubate at RT for an additional 10 min. Finally, the cells were again washed twice with PBS, and IBIDI liquid mounting medium (IBIDI) was added to the wells.

Cell images were then acquired with a LEICA SP5 confocal microscope (Leica Microsystems, Wetzlar, Germany) with a 40×/1.3 oil-immersion objective lens. Z-steps of 0.4 μm were used during acquisition for orthogonal view analysis.

### Statistics

GraphPad Prism 9 was used for graphical representation of results and statistical analysis. Tests used for the calculation of statistical significance are described in the corresponding figures. Results with *p* < 0.05 were considered statistically significant.

## Results

### In Silico Design

For developing the caged-siRNA nanostructure incorporating multiple siRNA sequences (in this specific design, three identical siRNAs are present), the design was based on two fundamental building blocks. The initial building block was based on a commercially available trebler phosphoramidite branching unit (https://www.glenresearch.com/10-1922.html). This building block is incorporated during DNA synthesis and allows to obtain a four-armed DNA dendron. The DNA arm 3′ of the trebler consists of a unique sequence, while the three other arms 5′ of the trebler all possess the same sequence (as they are synthesized simultaneously). This results in the formation of a DNA dendron type of structure (Figure 1A). The second building block comprises units of DNA–RNA hybrid sequences (henceforth designated D–R strands) with the RNA regions encoding the RNAi active sequences. The DNA region is complementary to the three identical DNA arm sequences in the 4-armed DNA dendron. Therefore, any oligonucleotide can theoretically be incorporated into this structure by complementary base pairing with the DNA arm sequences.

**Figure 1:**
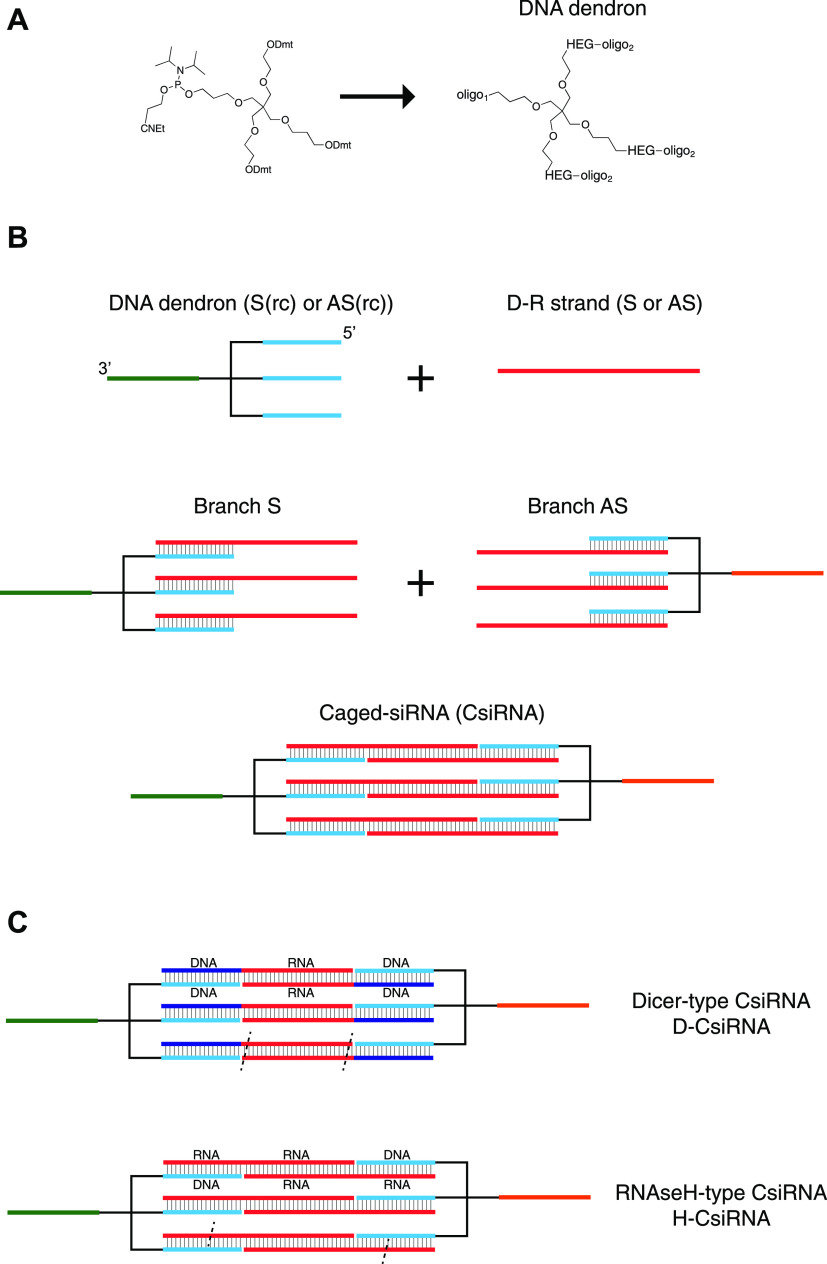
Schematic diagram of the steps leading to the formation of the basic caged-RNAi structure. (A) Trebler phosphoramidite is the building block allowing the synthesis of the initial DNA dendron type of unit. (B) Two-step assembly process of the CsiRNA begins with the separate assembly of the respective DNA dendron with its complementary D–R strand containing the sense or antisense RNAi sequence region. This forms the designated Branch structures. Afterward, the two Branch structures (S and AS) are combined in solution to self-assemble into the closed caged nanostructure. (C) Two types of structures studied comprise sequences that would support Dicer recognition-mediated release of the siRNAs (D-CsiRNA) or RNAse H-mediated release (H-CsiRNA). Dashed lines are an example of hypothetical regions of recognition and enzymatic cut by both Dicer or RNAse H, according to the specific design.

Finally, the assembly of the caged-siRNA nanostructure is a two-step process. First, through the annealing of three single-stranded D–R strands to the three complementary arm sequences in the DNA dendron, an intermediary structure is formed, designated as Branch (Figure 1B).

By performing this assembly with two different DNA dendrons, as well as two partially complementary single-stranded DNA/RNA hybrid oligonucleotides (D–R strands), the “sense” and “antisense” RNAi branches are obtained. These two structures are then assembled into a cage-like structure (closed structure) through hybridization of the respective complementary single-stranded RNA region. The RNA duplex formed can be designed *in silico* to correspond to an active siRNA sequence, thus forming a gene-specific caged-siRNA (CsiRNA) (Figure 1B).

To obtain the sequences giving the highest probability to form the designated intermediate and final structures, the NUPACK webserver^18^ was used. The gene-specific siRNA sequences were the only previously defined sequences used as input.

We evaluated two anti-GFP sequence designs based on a Dicer substrate-type siRNA sequence and its respective canonical 20/21bp siRNA sequence,^19,20^ as well as an anti-PTEN Dicer substrate-type siRNA based on ref (21).^21^

In the first design, the three DNA arms of the DNA dendron hybridize to complementary DNA sequences of the D–R strands (sense or antisense) carrying longer Dicer-type RNAi sequences. Dicer processing of the RNAi active regions would then lead to release the active siRNA sequences.

In the second design, the three DNA arms of the DNA dendron hybridize to a complementary DNA–RNA hybrid sequence of the D–R strands effectively forming regions of DNA–RNA duplexes that can be processed intracellularly by RNAse H. The action of the RNAse H would promote determined patterns of cleavage of the assembly arms leading to the release of the central RNAi active sequences (Figure 1C). Examples of the full sequences of assembled Dicer-based and RNAse H-based CsiRNAs (respectively, D-CsiRNA and H-CsiRNA) against GFP and anti-PTEN D-CsiRNA are shown in Figure S1.

### Assembly of “Dicer-Type” Caged-siRNA Structures

**Figure 2:**
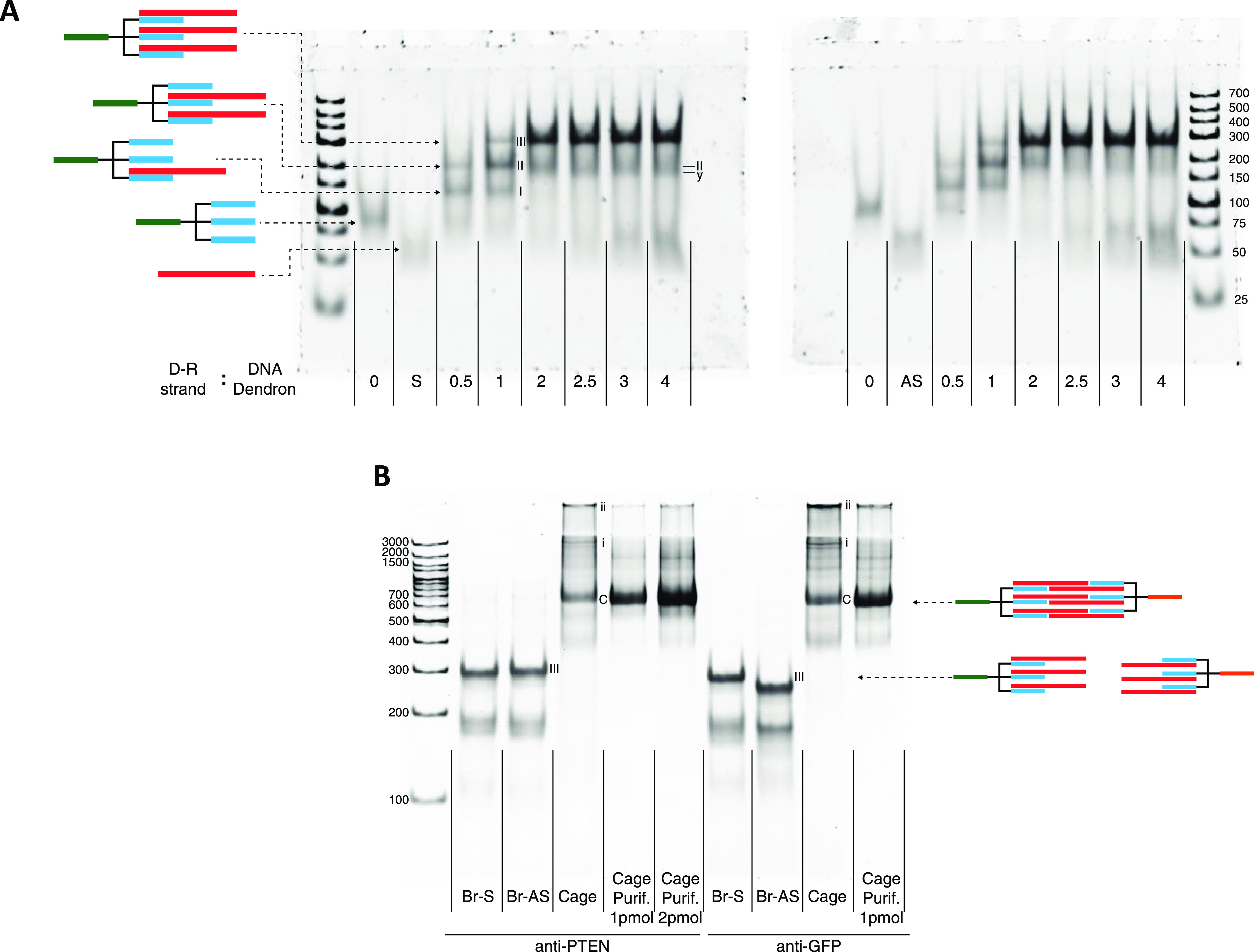
Polyacrylamide gel electrophoresis analysis of the D-CsiRNA assembly process with two RNAi sequences corresponding to anti-GFP and anti-PTEN. (A) Example of the first assembly step of the DNA dendrons with their respective D–R strand S or D–R strand AS of the anti-GFP RNAi sequence. Increasing molar amounts of D–R strand were added to the DNA dendron building block until the three available arms of the DNA dendron were fully occupied. This forms the designated Branch Sense or Antisense structure. The titration also shows the intermediate structures with one (I), two (II), or three (III) sites occupied. A small number of additional structures identified with “y” could also be observed that could correspond to some degree of intramolecular folding or interactions between strands in a single Branch. (B) Second assembly step involving the annealing between Branch S (Br-S) and Branch AS (Br-AS). This results in the preferential formation of a higher-molecular-weight band attributed to the formation of a closed structure with the interlocking of the three complementary strands from both Branch units (band denoted by the letter “C”). Some additional higher-molecular-weight bands with much lower proportion can be seen that can correspond to higher number (>2) of Branch S and AS units forming a closed structure (e.g., band “I”) or larger concatemers of several Branch units (e.g., band “ii”) that are stuck in the well. The cage band (“C”) can be isolated and gel purified with no disruption of its migration in the gel, thus, with no apparent alteration of the primary structure.

As stated above, the assembly process of CsiRNAs followed a two-step approach. The first assembly involved the separate annealing of each of DNA dendrons 1 and 2 with the corresponding complementary D–R strands carrying the sense and antisense RNAi active sequences. In this process, we typically used a thermal ramp involving a fast denaturation step at 85–95 °C followed by fast decrease to a fixed intermediate temperature with longer incubation time and final decrease of temperature to 4 °C. This formed the Branch S and Branch AS structures. This initial assembly process was followed by polyacrylamide gel electrophoresis for characterization of the resulting Branch structures (Figure 2A). The calculations of the extinction coefficients for both the DNA dendrons and the D–R strand (DNA–RNA) hybrid sequences is normally not completely accurate due to the size and complexity of the sequences and structures involved. Effects of hypochromicity due to formation of complex secondary structure regions (especially strong in RNA structures) are to be expected.^22^ As such, to find the most correct ratio at which all of the DNA dendron arms are occupied by the respective D–R strands, a titration with increasing ratios of D–R strand to DNA dendron was normally performed. With the increase in ratio of D–R strands, the occupation of the three complementary DNA arms is noticeable by the appearance of bands with increasing molecular weights and hence slower migration (the remaining DNA arm is left unhybridized as it has no sequence complementarity). Three bands with slower migrations in relation to the DNA dendrons are clearly visible, indicating the specific hybridization with one, two, or three D–R strands (respectively, band I, II, III annotated in the gel image). At optimal ratios, the DNA dendron bands are completely shifted, with tendency to form the highest-molecular-weight band III (three arms occupied). In addition to the noticeable formation of an intense band corresponding to 3× DNA arms occupied (band III), a lower band (band “y”) continues to be present even in the presence of excess of the corresponding D–R strand. This band could represent the DNA dendron remaining with only two DNA arms occupied. However, it is observable that it migrates with a slight difference corresponding to the DNA dendron with two arms occupied (band II). This implies that the band could correspond to DNA dendrons with the arms fully occupied but forming intra- or intermolecular secondary structures enabled by the flexibility of the RNA regions of the D–R strands. Analysis of the possible structures by NUPACK webserver (www.nupack.org) points to the possibility of intramolecular folding of the single-stranded RNA regions or intermolecular interactions between closely spaced RNA strands that could form regions of dsRNA (Figure S2). These folded structures, despite having the same molecular weight, can have a different migration pattern in PAGE due to structural differences.

The final assembly step involved the annealing of an equimolar amount (typically at 100 nM final concentration) of Branch S and Branch AS using a thermal ramp starting at a fixed intermediate temperature (typically 40–60 °C for 0.5 to 1 h) followed by a slow cooling step to room temperature and finally decreasing to 4 °C for storage if needed. This final assembly step was then verified by PAGE for characterization of the resulting CsiRNA (Figure 2B). A preeminent band (band “C”), with slower mobility compared to the Branch structures, is clearly formed together with some lesser visible higher-molecular-weight bands (bands i and ii) and a smear pattern that can correspond to misassembled or concatemer structures.

To drive the intramolecular annealing of the two Branch (S and AS) structures, it is important to optimize the concentration at which the incubation step is performed, as it is expected that low concentrations favor the formation of the closed caged-siRNA. When using different annealing concentrations ranging from 25 to 400 nM, it was visible that the Cage band (band “C”) was formed with a slightly lower yield when increasing the concentration. In addition, an increase in the proportion of larger aggregate structures (band “ii”), that are not able to migrate into the gel, is visible in the wells, and occur at the highest concentration used (400 nM) (Figure S4A). As expected, also, the assembly temperature was found to contribute to the yield of the assembly process. The temperature of incubation of the two Branch S and AS was determined based on a theoretical melting temperature (Tm) calculated for the duplex formed between the DNA dendron arms and the D–R strands, so as not to dissociate the already formed Branch structures. However, due to the inherent multimeric nature of this structure and possible cooperativity effects between oligonucleotide arms of identical sequences, these Tm should be considered only as rough approximations. An optimization of the process can thus be attempted by incubations at different temperatures (Figure S4B) where it can be observed that the proportion of the aggregate band (band “ii”) of very high molecular weight, and also of some lower-molecular-weight bands (band “s”), varies depending on the temperature.

To isolate the correct CsiRNA structure for further work, gel purification methods were used. The recovered material was mostly free from larger aggregates and concatemers and the structures were shown not to suffer any alteration after the process, as verified by PAGE (Figure 2B).

Ultimately, the final optimized assembly yield of the “Dicer-type” CsiRNA, calculated by gel band quantification after PAGE, was 23 ± 2% (average calculated from four independent assemblies ± standard deviation).

**Figure 3:**
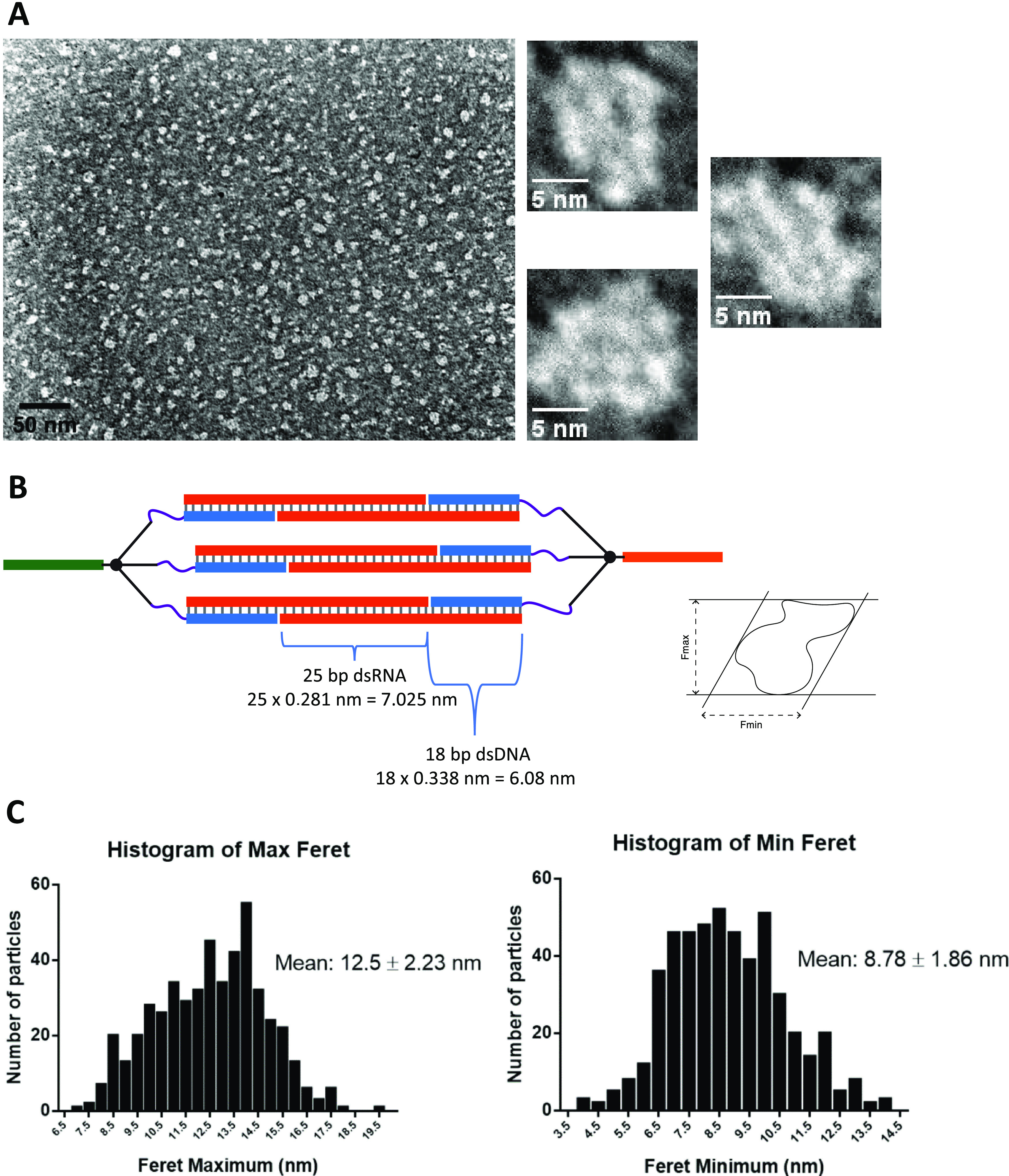
Structural characterization of purified CsiRNAs through transmission electron microscopy (TEM). (A) Typical negative-staining TEM micrograph and examples of individual particles obtained through semiautomatic picking using the Xmipp3 plugin. (B) Schematic representation of the caged-siRNA structure with the annotated dimensions used for size estimations. Picture inserted to the right represents the definition of maximum Feret (Fmax) and minimum Feret (Fmin) for the analyzed objects. (C) Histogram of Ferret diameter measurements for individual particles (*n* = 496 particles from four different micrographs).

To further confirm that band C corresponded to discrete molecular structures, we analyzed the purified band by TEM (Figure 3). As the structure has many points of increased flexibility, such as the connecting ethylene-glycol linkers and the single-strand nicks (or nonligated), at specific points of the double-strand regions, it is conceivable that it can adopt different shapes during adsorption to the TEM grids. Observation of the purified sample by TEM demonstrated the presence of discrete structures complying with maximum dimensions (longest axis) up to *ca.* 20 nm and average dimensions of *ca.* 12–13 nm (longest axis). Taking into consideration the following factors: (a) the negative-staining TEM protocol used would allow preferential observation of double-stranded DNA/RNA regions; (b) the central double-stranded region of the cages contain noncontiguous strands (nonligated/nicked) that cancel the rigidity of the double-stranded helix and allow high flexibility at those points; (c) the structures are deposited onto a cationic surface and dried down; (d) distance length between base pairs of DNA of 0.338 nm and RNA of 0.281 nm; we observed that the experimentally obtained dimension values are in conformity to the overall theoretical dimension estimates calculated based on the cage design (see Figure 3).

### Design and Assembly of “RNAse H-Type” Caged-siRNA Structures

**Figure 4:**
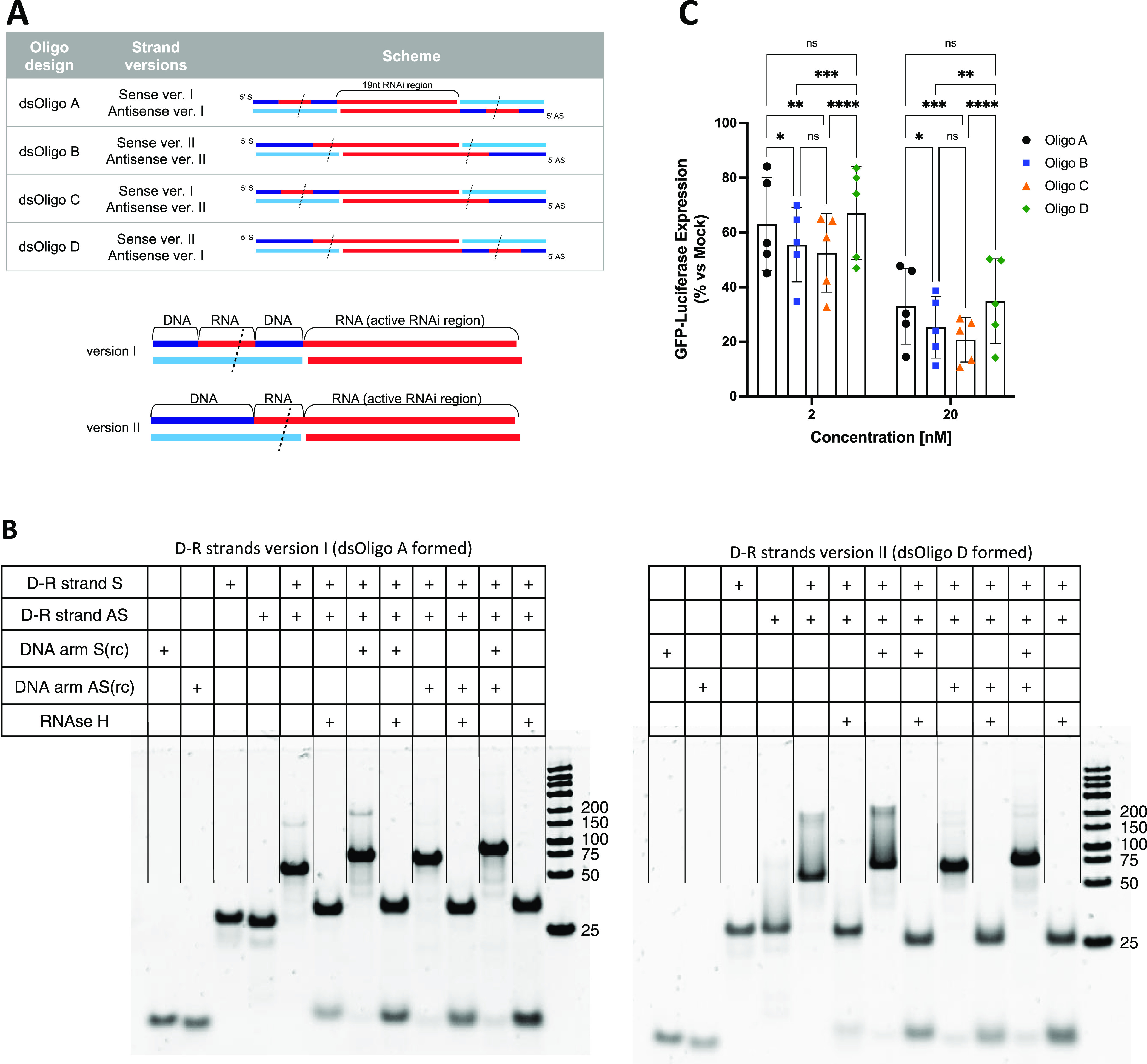
Design and in vitro testing of D–R strands for RNAse H recognition. (A) Complementary regions to the DNA dendron arms of the D–R strands (S and AS) were designed to have either a DNA–RNA–DNA or a DNA–RNA design that can, once annealed to the DNA dendron arms, form a hybrid DNA–RNA double-stranded region. These are formed to elicit the recognition and cleavage by RNAse H. Different combinations of these two designs formed different versions of dsOligos to test. (B) PAGE analysis of *in vitro* RNAse H cleavage assay of the dsOligo design versions. (C) Silencing activity of the dsOligo versions 48 h after *in vitro* transfections. Columns represent average values normalized to mock transfections ± SD. Statistical significance evaluated by repeated-measures two-way ANOVA with Tukey post hoc tests (*p < 0.05; **p < 0.01; ***p < 0.001; ****p < 0.0001).

To assemble the caged-siRNA structure for RNAse H-mediated release of the central active RNAi region, two different linking arm sequences for the D–R strand S and D–R strand AS were designed. Hence, the complementary regions of the D–R strands (Sense and Antisense) to the DNA dendron arm were designed to have a DNA–RNA–DNA block (version I) or a DNA–RNA block (version II). This created four different design versions as depicted in Figure 4A. In addition, the RNAi region was shortened to resemble a canonical siRNA of 19 bp, thus abolishing the prerequisite length for Dicer recognition (see Figure S1). The recognition and processing of the designed oligos by RNAse H were tested by assembly of the dsOligos. DNA sense arms and DNA antisense arms were used to hybridize to the single-strand extensions of the D–R strands, mimicking the double-stranded region when connected to the DNA dendron arms, as illustrated in Figure 4A. The assembled dsOligos were then incubated with recombinant RNAse H *in vitro*. PAGE analysis of the resulting products allowed us to confirm that all of the versions elicited RNAse H activity with the release of the central double-stranded region, demonstrated by the formation of specific lower-molecular-weight bands and release of the corresponding complementary DNA arms at the 5′ and 3′ ends (Figure 4B). Notably, the annealed D–R strands (S–AS) without the corresponding complementary DNA arms are also processed by RNAse H with the formation of a specific faster migrating band. By posterior *in silico* folding analysis, we found that it is possible for the flanking single-stranded ends to form hairpin structures where the hairpin stem contains a small DNA–RNA hybrid double strand, which can in principle be recognized by RNAse H (Figure S3). The different dsOligo design versions (containing the RNAi regions against GFP) were then transfected to test for any differences in their activity (Figure 4C). There was an improved activity for both constructs (dsOligo B and C) having the AS strand with the version II design (where the RNA region being cut is placed immediately 5′ to the active AS sequence). This showed that a DNA nucleotide extension 5′ to the AS sequences can have a slight detrimental activity. Although not statistically significant, there was a tendency for dsOligo C design to have a slight improved silencing activity over dsOligo B. This design was then used to assemble the full H-CsiRNA structure.

The assembly and purification of the H-CsiRNA cages, with the dsOligo C design, proceeded similarly to the previous D-CsiRNA and was characterized by PAGE as before (Figure S5). Ultimately, the final optimized assembly yield of the “RNAse H-type” CsiRNA, as calculated by gel band quantification after PAGE, was 27 ± 5% (average calculated from four independent assemblies ± standard deviation).

### In Vitro Enzyme (Dicer and RNAse H) Processing of CsiRNAs

**Figure 5:**
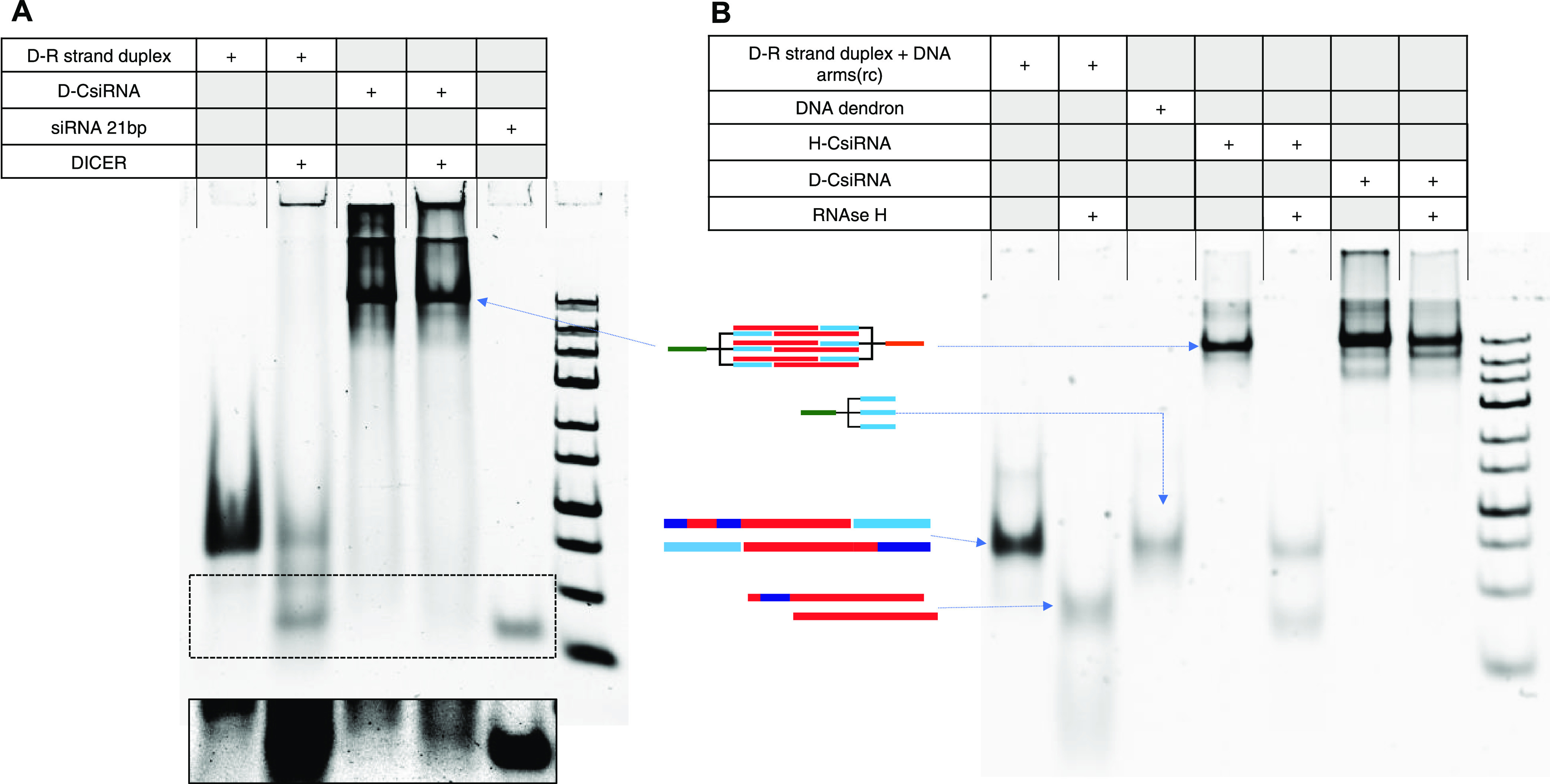
Cleavage of CsiRNAs by Dicer and RNAse H. (A) Native PAGE stained by SYBRGold showing the D-CsiRNA structure incubated with recombinant Dicer enzyme for 6 h. The core region of the CsiRNA containing the hybridized D–R strands S and AS was used as a cleavage control. An siRNA targeting the same GFP region as the CsiRNA was used as a size marker for the gel run. The boxed region is shown below the gel with an enhanced contrast setting. (B) Native PAGE gel stained with SYBRGold showing the H-CsiRNA structure incubated with RNAse H. The D-CsiRNA was used as the negative control for RNAse H recognition. As before, the hybridized D–R strands (S–AS) were used as controls for generation and identification of the RNAse H cleavage fragments.

As a preliminary analysis of the potential recognition of the D-CsiRNA structures by Dicer enzyme, an *in vitro* cleavage assay using recombinant Dicer was used (Figure 5A). After incubation, the structures were loaded on a polyacrylamide gel to resolve the resulting fragments. There was, however, a low *in vitro* activity of the Dicer enzyme on the D-CsiRNA structures. This was observed by the persistence of a significant amount of the intact structure band and the appearance of a residual amount of a lower-molecular-weight band with a size corresponding to the cleavage product of the control double-stranded D–R strand S–AS (see also Figure S6).

For the processing of H-CsiRNAs, the structures were incubated *in vitro* with RNAse H and samples run by PAGE. In Figure 5B, it is clearly observed that H-CsiRNA are easily cleaved by RNAse H in a specific process that results in the release of the subcomponents forming the closed caged structure, namely, the DNA dendron and the processed central dsRNAi sequence. On the other hand, the D-CsiRNA version was completely resistant to RNAse H incubation.

### In Vitro Activity

Both purified CsiRNAs were then tested for their capacity to downregulate the corresponding gene *in vitro* after transfection.

**Figure 6:**
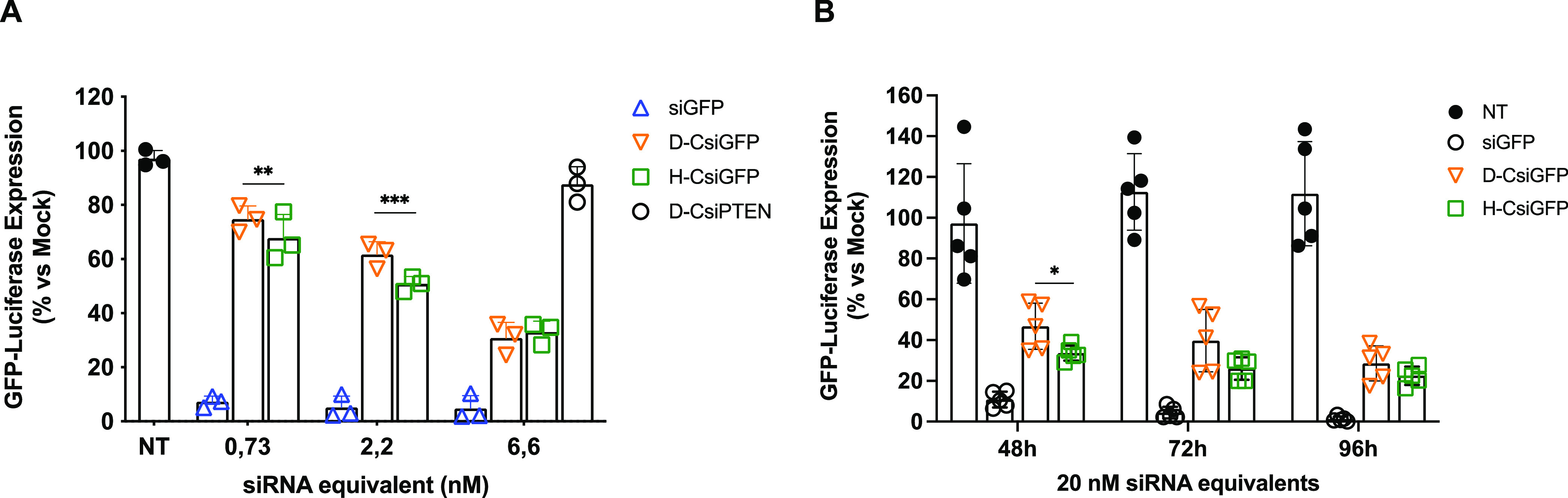
Luciferase assays of U2OS-GFPLuc transfected with CsiRNAs. (A) Cells transfected with different concentrations of the two versions of CsiRNA and analyzed 96 h post-transfection. A D-CsiRNA against PTEN gene was used as a negative control. Columns represent average values normalized to mock transfections ± SD. Statistical significance evaluated by repeated-measures two-way ANOVA with Tukey post hoc test (*n* = 3 independent experiments; **p < 0.01). (B) Cells transfected at 20 nM concentration and analyzed at different post-transfection time points. Columns represent average values normalized to mock transfections ± SD. Statistical significance evaluated by one-way ANOVA independently for each time point, with Tukey post hoc test (*n* = 5 independent experiments; *p < 0.05).

When the U2OS-GFPLuc cells were transfected at progressively higher concentrations of the cages and analyzed at the 96 h time point, there was an increase of the gene silencing efficiency reaching around 70% at 6.6 nM for both types of structures (Figure 6A). Interestingly, although the overall siRNA structure dramatically departs from classical synthetic canonical siRNA or Dicer-siRNA structural features, we were indeed able to maintain the gene silencing activity. There seems to be a tendency for the H-CsiRNA version to perform slightly better than the D-CsiRNA albeit, only at the 2.2 nM concentration the difference was found to be statistically significant. Importantly, a D-CsiRNA with an unrelated sequence (PTEN) did not show a significant silencing activity in this assay, thus pointing to the specificity of the structures.

The CsiRNAs were also transfected at a single concentration (20 nM) and activity measured at different time points ranging from 48 to 96 h (Figure 6B). Under these experimental conditions, the silencing activity increased from 48 to 96 h, reaching a maximum silencing close to 80%. At earlier time points, the H-CsiRNAs seem to be more efficient than D-CsiRNAs, possibly pointing to the fact that the release of the active RNAi sequences from the caged structures can be more efficient if dependent on RNAse H than through Dicer recognition.

### In Vitro Activity of H-CsiRNAs with Extensive Chemical Modification Patterns

It has been recognized that for improving the efficiency of the siRNA silencing activity *in vivo*, the use of extensive chemical modifications of the siRNA molecule is advantageous, especially in the case of siRNA conjugates.^23,24^ Our construct would be especially suited for conjugate delivery, and as such, we set out to test its efficacy when heavily modifying the sense and antisense strand regions.

**Figure 7:**
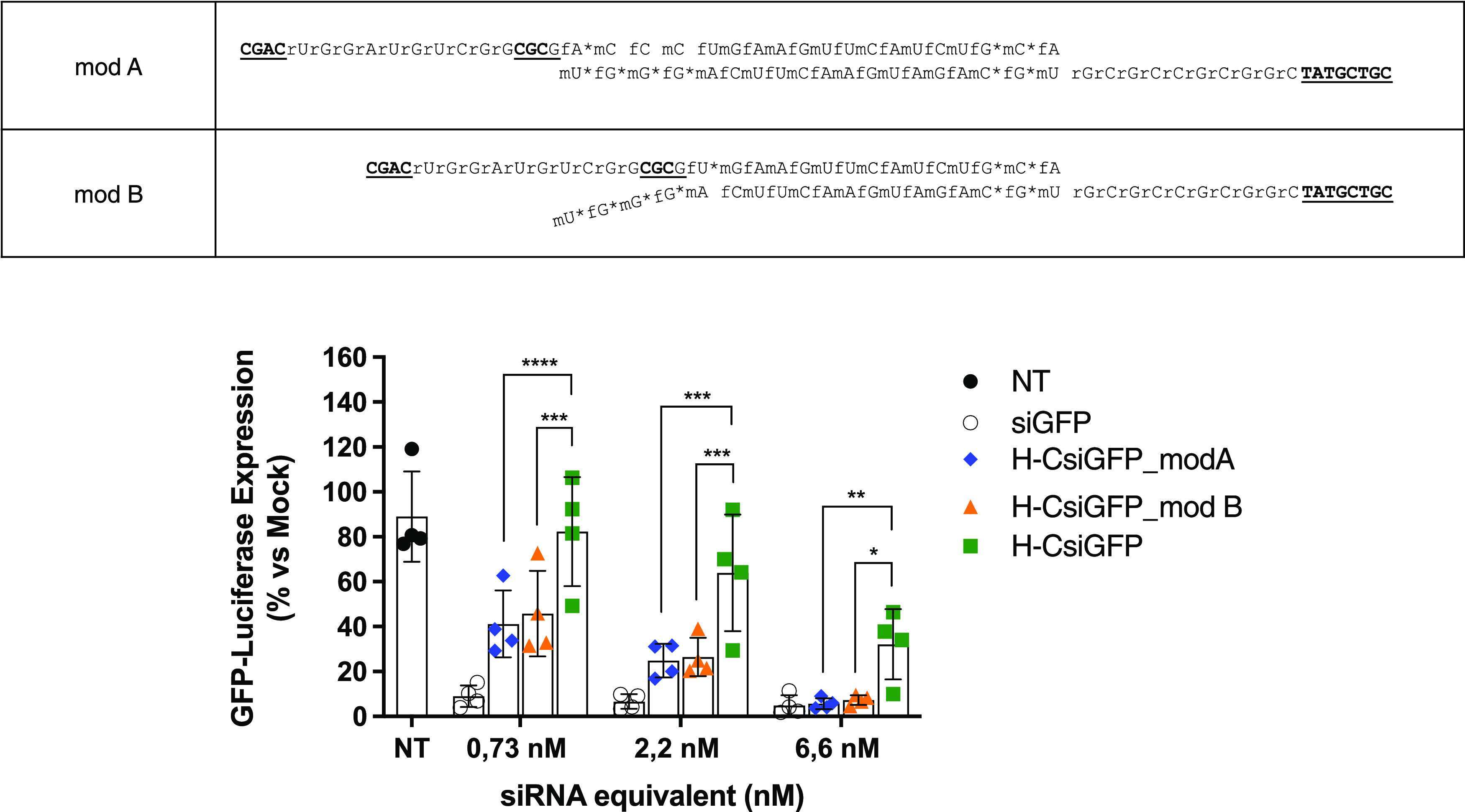
Luciferase assay of U2OS-GFPLuc cell transfected with extensively modified H-CsiRNAs. Cells transfected with different concentrations of the different versions of H-CsiRNA and analyzed at 96 h post-transfection. Above the graph, the two modified versions of D–R strands (S and AS) are shown, with the complementary nucleotide pairing, that compose the H-CsiRNA_modA and _modB versions. The notations represent fN = 2′ fluoronucleotides, mN = 2′*O*-methyl nucleotides, **N (bold, underlined) =** DNA nucleotides, rN = RNA nucleotides. The graphical results represent mean values normalized to mock transfections ± SD. Statistical significance evaluated by repeated-measures two-way ANOVA with Tukey post hoc tests (*p < 0.05; **p < 0.01; ***p < 0.001; ****p < 0.0001).

For this, we chose an alternate pattern of 2′*O*Me and 2′F modifications in both the sense and antisense (cross-pattern, Figure 7). In addition, we tested a truncated version of the sense region (15 bases), in similarity to the asymmetry concept.^25,26^ The final optimized assembly yield of the modified “RNAse H-type” CsiRNA was calculated by gel band quantification after PAGE and was found to have a value of 28 ± 2.5% (average calculated from three independent assemblies ± standard deviation), thus not departing from the values found for the unmodified “RNAse H-type” CsiRNA.

When modifying the previous H-CsiRNA, we could effectively verify a more potent silencing by both regular and asymmetric versions after transfection (Figure 7), with similar effect to a canonical siRNA at 6.6 nM concentration (the highest concentration tested in terms of siRNA molecules transfected).

### Cellular Activity of Functionalized Caged-siRNA Structures

The proposed nanostructured CsiRNA presents in this version two functionalization handles (ssDNA anchors) (see Figure 1B). These can be utilized for the flexible functionalization of the structures with different biological moieties to achieve, for example, enhanced cellular uptake. Here, as a model ligand, we have used a peptide–DNA conjugate, using the Tet1 peptide specific for the neuronal trisialoganglioside cell receptor Gt1b,^27^ as well as a Cy5-labeled ssDNA strand with a fully phosphorothioate 6-nucleotide extension tail (_Cy5_PStail). Both ligands thus present a DNA handle that is specifically hybridized to one of the anchors present in the CsiRNA.

**Figure 8:**
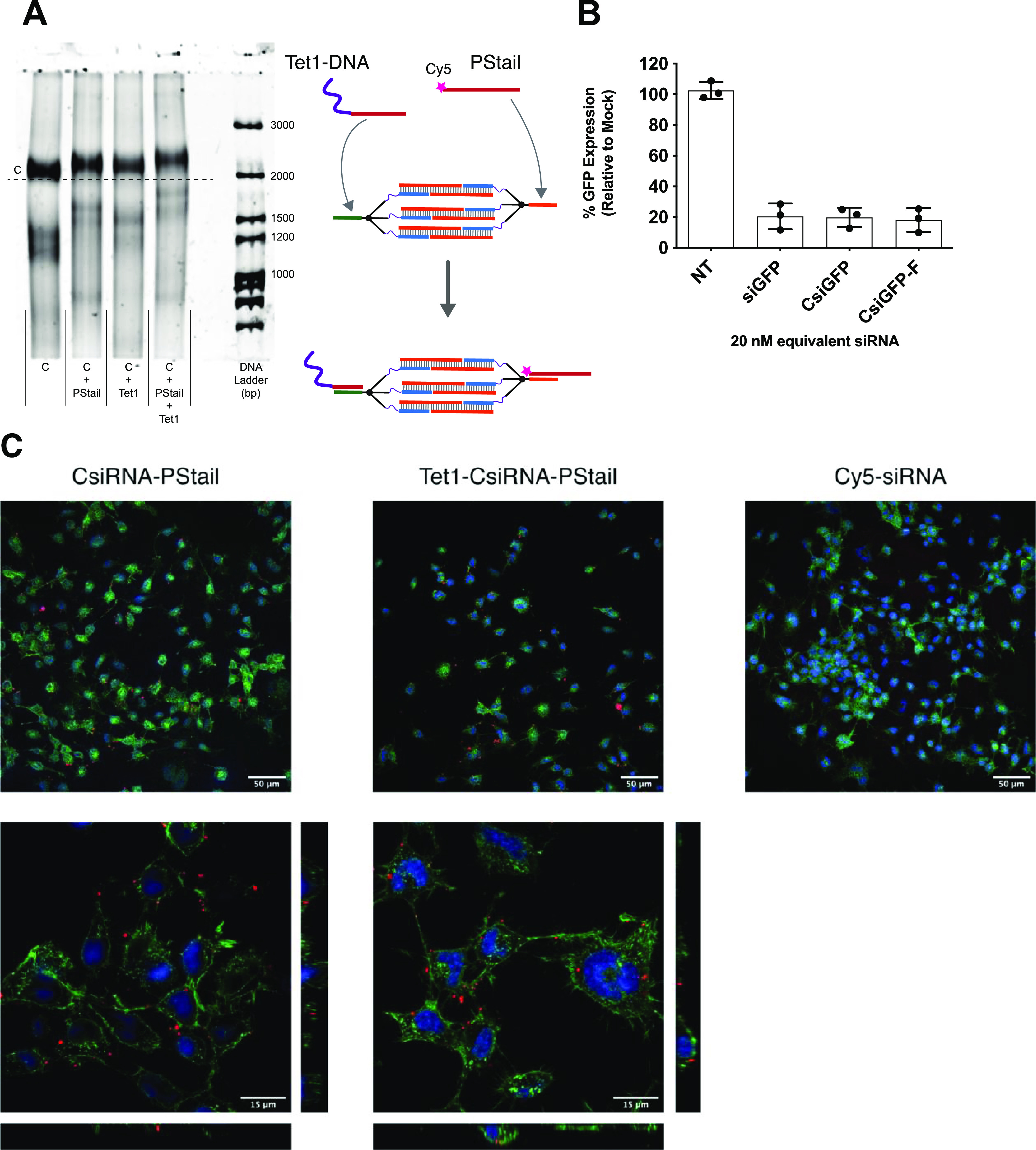
Cellular activity of functionalized CsiRNA structures. (A) Native PAGE showing the hybridization of CsiRNAs with the DNA-Tet1 conjugate and a fluorescently (Cy5) labeled phosphorothioated short oligo tail (PStail) to the respective anchor sequences. The assembly process is schematically represented to the right of the gel image. (B) Silencing activity of the functionalized CsiRNAs-F (Tet1-CsiRNA-_Cy5_PStail) after *in vitro* transfection, as evaluated by flow cytometry analysis of median cell GFP fluorescence. (C) Representative micrographs of the cellular association of Cy5-labeled siRNA versus CsiRNA-_Cy5_PStail and Tet1-CsiRNA-_Cy5_PStail (without lipid transfection reagent used). Nuclei are represented in blue, membrane staining in green, and both CsiRNA and siRNA fluorescence are represented in red. The upper panel shows representative general confocal microscopy photos with Max Z projections and the lower panels show orthogonal views, at 4x scale, corresponding to a confocal slice passing through the middle of the cell (Cy5-siRNA did not show a fluorescence signal and thus is not represented).

By a simple annealing procedure, we could verify by PAGE analysis the effective functionalization of the CsiRNA structures with each ligand (Figure 8A) shown by a shift in the mobility of the CsiRNA in the gel (increase in size).

We then proceeded to verify if these additional moieties would affect the overall gene silencing activity of the CsiRNA. Using the same in vitro cell transfection assays as above, to focus on the intracellular effects of the structure, we could verify no significant alteration of the gene silencing capabilities of the CsiRNA (Figure 8B). This demonstrated the flexibility of our functionalization strategy that, along with the programmed release of the RNAi regions from the CsiRNAs, allows to maintain the intrinsic efficacy of silencing, independent of the ligand attached.

Finally, we verified if there would be additional cell–CsiRNA interactions afforded by the functionalization of the CsiRNAs and without the use of lipid transfection reagents. For this, we analyzed by confocal microscopy the cellular association, of Tet1-CsiRNA-_Cy5_PStail functionalized structures with a neuronal cell line ND7/23 (neuroblastoma cell type) known to express the neuronal trisialoganglioside cell receptor Gt1b^28^ (Figure 8C). Structures functionalized with CsiRNA-_Cy5_PStail, without the Tet1 ligand, and a Cy5-labeled siRNA were used as additional controls. Interestingly, under the chosen experimental conditions, we observed an increased association to cells of the CsiRNA constructs in comparison to an almost absolute absence of signal from a canonical Cy5-labeled siRNA. In this in vitro context, both CsiRNA-_Cy5_PStail and the Tet1-CsiRNA-_Cy5_PStail showed qualitatively similar association and localization in the cells, which was mostly at the membrane, with some fluorescence coming from internalized constructs.

## Discussion

The siRNA conjugate approach has been receiving much of the latest clinical focus with most siRNA drug candidates belonging to this category.^29^ This has been spearheaded by the GalNAc conjugate platform for liver targeting and the *de facto* demonstration of clinical utility coming with the approval of the GalNAc-conjugated siRNA drug, GIVOSIRAN.^30,31^ This success has renewed the interest in siRNA drugs and their development for indications outside liver targeting and potentially for common and complex diseases. While the nucleotide chemical toolbox is one way to address the challenges in siRNA delivery, structural molecular engineering could also play an important role. This can be acknowledged in recent work demonstrating improved central nervous system delivery by a divalent RNAi scaffold,^32^ or initial work demonstrating the possibility to integrate active Dicer-based RNAi sequences in more complex RNA origami 3D structures.^33^

Here, we have explored a novel simple structural scaffold, engineered through self-assembly of branched oligonucleotide units carrying multiple RNAi trigger sequences. The proposed design allowed the formation of a structure enclosing multiple siRNAs in its central core, which we have designated caged-siRNAs (CsiRNA). Two alternative design principles were used in the structure leading to the RNAi trigger. A first design explored the release of the RNAi trigger through the action of the Dicer enzyme by employing Dicer-type RNAi sequences, specifically a 27/27 blunt end design in the central region of the structure. The second design explored the release of the RNAi trigger through the insertion of an RNAse H-recruiting RNA–DNA duplex region flanking the central 19/21 (S/AS strand) canonical siRNA trigger. Both designs relied on a two-step self-assembly process with the initial building blocks being formed by DNA dendrons, synthesized using a trebler phosphoramidate, to which the corresponding extended sense and antisense strands would first anneal. These initial building blocks stemmed from previous strategies of constructing synthetic oligonucleotide dendrimers, which could assemble into a caged type of structure if two such units comprised complementary oligonucleotides attached to the trebler core through the same end (3′ end), as first described by Shchepinov et al.^11^ A stepwise assembly strategy was then devised to introduce multiple RNA sequences into the DNA dendrimeric (or DNA dendron) unit. This strategy was crucial to achieve a practical approach for the introduction of long RNA strands attached to the trebler unit. This was due to the foreseen complexity to attain this in a single DNA synthesis step and the subsequent follow-up purification that would be needed. This approach would also allow to interchange RNAi sequences on demand more easily. The second assembly step involved the subsequent hybridization between the DNA dendrons holding the complementary sense and antisense oligonucleotide sequences (designated by Branch). This assembly, forming the closed caged structures from two pairs of complementary units, would in theory be favored in opposition to the formation of concatemers. This is expected to occur due to a higher stability (or Tm) imparted by the local increase of concentration established by the presence of multiple arms of the DNA dendrons in proximity. This effect is potentiated in diluted solutions of both Branches during assembly. As expected (see Figure S4), an increase in the propensity to form aggregates is indeed observed with increasingly higher concentrations of Branches during assembly. In general, assembly yields of the presented Caged-siRNA design ranged from 25 to 30%. Taking into consideration the flexibility of the linking trebler units, these initial assembly yields are good. The preferential assembly of the closed structures should rely mostly on the cooperativity effects coming from the initial binding of the first arm. This in turn should locally increase the concentration of the remaining arms, thus also directly increasing the likelihood of the remaining intramolecular hybridizations to occur. As we observed, this is governed by a concentration, temperature, and ion dependent effect. Thus, we believe a detailed investigation into assembly conditions, as well as the use of less flexible linkers, should allow further increases in yields.

TEM imaging allowed us to confirm the presence of discrete structures after isolation of the gel bands corresponding to the expected closed caged-siRNAs. Theoretical calculations taking into account the known DNA and RNA dimensions allowed to estimate an average maximum length (longest axis) of the central double-stranded core region of the structure with the following considerations: (a) assuming a completely extended 2D plane projection of the structure (to more closely resemble the adsorption and drying onto the grid surface for electron microscopy imaging) and (b) assuming no flexibility from the nicked/nonligated strands in the double-stranded regions. Thus, a theoretical value of 19 nm (6 + 7 + 6 nm) for the longest axis can be calculated (Figure 3B). If we consider the width of the double-stranded DNA/RNA of *ca.* 2 nm, then three strands juxtaposed in parallel fashion would give a length of the smallest axis of 6 nm. However, there is an inherent flexibility to the macromolecule, which is expected due to the presence of ethylene-glycol-based linkages on the trebler unit and the noncontinuous nature of the double-stranded central core sequence region. The longest length of the central dsRNA with no single-strand breaks would result in the long axis having 7 nm (Figure 3B) in a total extended configuration. For TEM imaging, the nucleic acid-based structures were adsorbed to a PDL-coated grid and dried down. These, being flexible macromolecules, can deposit in the grid in multiple irregular conformations. For this reason, we used a semiautomated method to pick the particles and calculated average maximum and minimum Ferret’s diameters (defined as the distance between two parallel planes restricting the object perpendicular to that direction). The obtained sizes of *ca.* 12–13 and 9 nm for the maximum and minimum Ferret’s diameter, respectively, indicate a close conformity to the theoretically predictable sizes of a single caged-siRNA macromolecule unit comprising the three linked RNAi regions. In addition, we could observe for some objects particular features indicative of the presence of three dsRNA strands held in proximity.

We have subsequently unveiled that one can specifically control the release of the RNAi triggers from the self-assembled nanoarchitecture, especially when using the newly developed RNAse H-powered disassembly mechanism. Our first design made use of Dicer recognition RNAi sequences with extended sequences of 27/27 bp (S/AS). In *in vitro* conditions, we were only able to detect a very residual fraction of shorter siRNA sequences when incubating the Dicer-based cages with recombinant Dicer enzymes. In contrast, the RNAse H-based cages did show a complete disassembly of the structure and release of shorter dsRNA sequences *in vitro*. After transfections, the RNAse H cages showed a slight enhancement of silencing activity, overall, versus Dicer cages (Figure 6). We also noticed that depending on the exact design of the RNA–DNA hybrid, we could induce the release of dsRNA fragments with a slightly different gene silencing activity (Figure 4C). The designs that, after RNAse H processing, left antisense strands with 5′ DNA extensions had reduced activity in comparison to 5′ RNA extensions. This goes in line with the previously demonstrated less tolerance of the 5′ end of the antisense for modifications and/or conjugation of molecules at this end.^34−37^ Nevertheless, it should be noted that an RNA extension is also not optimal as the overall effect on gene silencing efficacy, when compared with a canonical siRNA, is decreased. Still, this shows that with careful design and further optimizations, the RNAse H could cut the sequences in optimized patterns to at least recover the full potential gene silencing activity and possibly also bringing an added level of control.

Of interest is the fact that the whole architecture supports the introduction of heavily modified sense and antisense sequences. We tried two designs, one using a 19/19 bp and the other an asymmetric design with 15/19 bp (S/AS), both using alternating 2′F and 2′*O*Me nucleotides, with terminal PS linkages. Both designs led to a significantly improved gene silencing potency of the caged nanoarchitectures, reaching >90% silencing after *in vitro* transfections at very low doses of 6,6 nM of total siRNA equivalents or corresponding to 2.2 nM of Caged-siRNA (CsiRNA). Interestingly, no significant differences were found between both designs after transfection, meaning the asymmetric design could be useful if a smaller size of the overall construct is needed. However, future experiments regarding *in vivo* accumulation in different organs could provide additional data when comparing both constructs.

Since the Caged-siRNA presents two anchor sites for further functionalization, we tested this feature using as example ligands a DNA-Tet1 peptide conjugate (with Tet1 as a previously identified peptide ligand to the trisialoganglioside receptors Gt1b) and a Cy5-labeled ssDNA with a fully phosphorothioated (PS) 6-nucleotide tail (PStail), respectively, for each available anchor site. The Tet1 peptide has been utilized in other systems to provide some increased specificity toward neuronal cells.^38^ The PStail was utilized in light of the known phosphorothioate (PS) effects regarding general enhancement of cell uptake of PS-modified oligonucleotides.^32,39,40^

Functionalization of each Cage anchor consisted of a simple process of annealing the ligands through their complementary DNA extensions (Figure 8). This results in a modular assembly that allows to easily assess other DNA-ligands of interest. Importantly, the functionalization process of the Caged-siRNA structure with the ligands did not alter its intracellular gene silencing efficiency. This can be attributed to the disassembly mechanism of the Cage by the RNAse H action, which releases the RNAi sequences, making them unsusceptible to the type and/or size of ligands attached.

Cellular interaction of the functionalized Caged nanostructures was assessed by incubation (without lipid transfection reagents used) with a neuroblastoma cell line (ND7/23) known to express the GT1b receptor. Through confocal microscopy, we observed that indeed the functionalization of the Cages could influence the association of the nucleic acid structure with the cells. Although under the *in vitro* conditions tested no significant increase of the internalization potential was attained, the interaction of the functionalized cages with the cell membranes was enhanced. This association was promoted similarly in the structures functionalized with the _Cy5_PStail and the ones doubly functionalized with _Cy5_PStail and Tet1, implying that Tet1 did not contribute, in this system, to significant improvements in cellular internalization. This could be attributed to an unexpectedly low binding affinity of Tet1 to the proposed Gt1b receptor as demonstrated more recently^41^ and not a feature of the structure itself. Although the apparent lack of significant effect of the ligands explored in this study can be seen as a limitation, the conceptual framework of our proposed structure, with inbuilt modularity, allows for a quick and easy interchange between functional ligands, as demonstrated. Thus, other biological active ligands can easily be probed in future work.

## Conclusions

Ultimately, in this work, we present a conceptual design using self-assembly of multiple RNAi trigger sequences, which integrate the nanoarchitecture scaffold while containing their own independently controllable disassembly mechanism. We propose that using RNAse H sequence recognition cues can enable the controlled release of RNAi active regions, a method that can be applied to many different types of DNA/RNA nanostructures bearing siRNAs in their framework. This design does not use extensions per se for integrating RNAi sequences in an RNA/DNA scaffold structure, leaving such anchor sequences dedicated exclusively for further functionalization with added biological moieties (e.g., for cell targeting purposes).

Our single RNAi molecular construct allows the exploitation of the following important features: (1) Entry of multiple siRNA molecules per single cell uptake event through receptor-mediated endocytosis. This would allow to increase the efficacy of siRNA delivery when using receptors of lower efficiency when comparing to the demonstrated asialoglycoprotein receptor in hepatocytes;^30^ (2) Easy functionalization with multiple homo or heteroligands for exploitation of different organ/cellular delivery mechanisms.

In the future, the generalization of the presently described mechanisms together with adjustments to sequence design, choice of linker, and geometry, can allow the introduction of multifunctionality. Here, the use of different siRNA sequences (targeting different genes) would enable simultaneous and synergistic action, which can be potentially important when dealing with complex diseases.

Overall, the presented features can be adaptable to improve other DNA–RNA-based nanoarchitectures as nucleic acid delivery vectors.

## Supporting Information Available

Schematic drawing and sequences with representation of nucleotide base pairing of the original D-CsiRNA and H-CsiRNAs against GFP (Figure S1); thermodynamic intramolecular folding and intermolecular strand interaction analysis of sense and antisense A D–R strand (GFP) sequences (Figure S2); thermodynamic intramolecular folding analysis of the DNA Dendron arm complementary binding sequences of the sense and antisense D–R strands versions I and II (GFP) (Figure S3); polyacrylamide gel electrophoresis analysis of the second step in the CsiRNA assembly process (Figure S4); polyacrylamide gel electrophoresis analysis of the H-CsiRNA assembly process and purification (Figure S5); and additional polyacrylamide gel electrophoresis analysis of the processing of D-CsiRNA by recombinant DICER (Figure S6) (PDF)
