# Supplementary material for: Engineering a Novel Self-Assembled Multi-siRNA Nanocaged Architecture with Controlled Enzyme-Mediated siRNA Release
Source: ACS Appl Mater Interfaces. 2022 Dec 15;14(51):56483–97. doi: 10.1021/acsami.2c15086 (PMC9801385; doi:10.1021/acsami.2c15086)
Supplement: Supplementary file 1 — am2c15086_si_001.pdf [file am2c15086_si_001.pdf]

SUPPORTING INFORMATION

Engineering a novel self-assembled multi-siRNA nanocaged architecture with controlled enzyme-mediated siRNA release

Pedro M.D. Moreno[*#†] , *João Cortinhas*[#†] , *Ana S. Martins*[#†‡] , *Ana P. Pêgo*[*#‡§]

# i3S - Instituto de Investigação e Inovação em Saúde, Universidade do Porto, 4200-135 Porto, Portugal

† INEB - Instituto de Engenharia Biomédica, Universidade do Porto, 4200-135 Porto, Portugal

‡ Faculdade de Engenharia da Universidade do Porto, 4200-465 Porto, Portugal

§ Instituto de Ciências Biomédicas Abel Salazar (ICBAS), Universidade do Porto, 4050-313 Porto, Portugal

* To whom correspondence should be addressed. Tel: +351 220 408 800; Email: pedro.moreno@ineb.up.pt; apego@ineb.up.pt

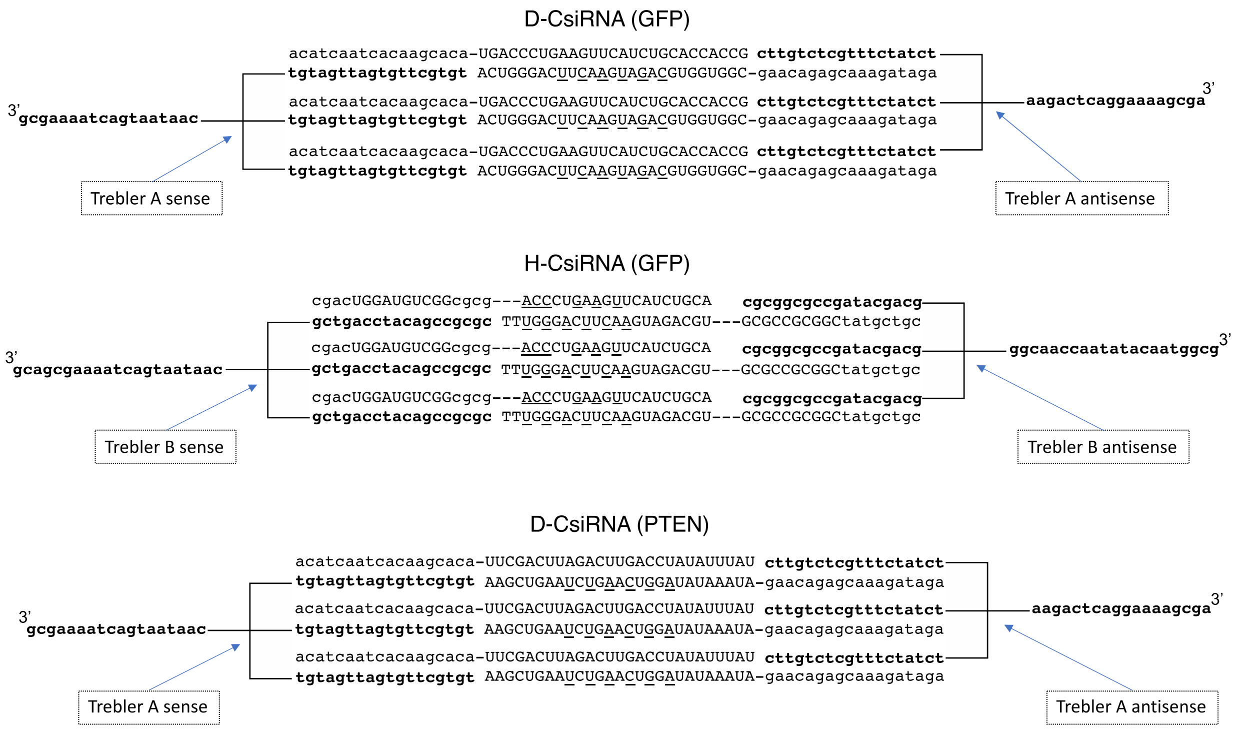

Figure S1. Schematic drawing and nucleotide sequences of the original D-CsiRNA and H-CsiRNAs against GFP. The different letter types represent respectively: DNA dendron bases = small bold letters (**n**); DNA bases = small letters (n); RNA bases = capital letters; 2'*O*Me-RNA = underlined capital letters (N).

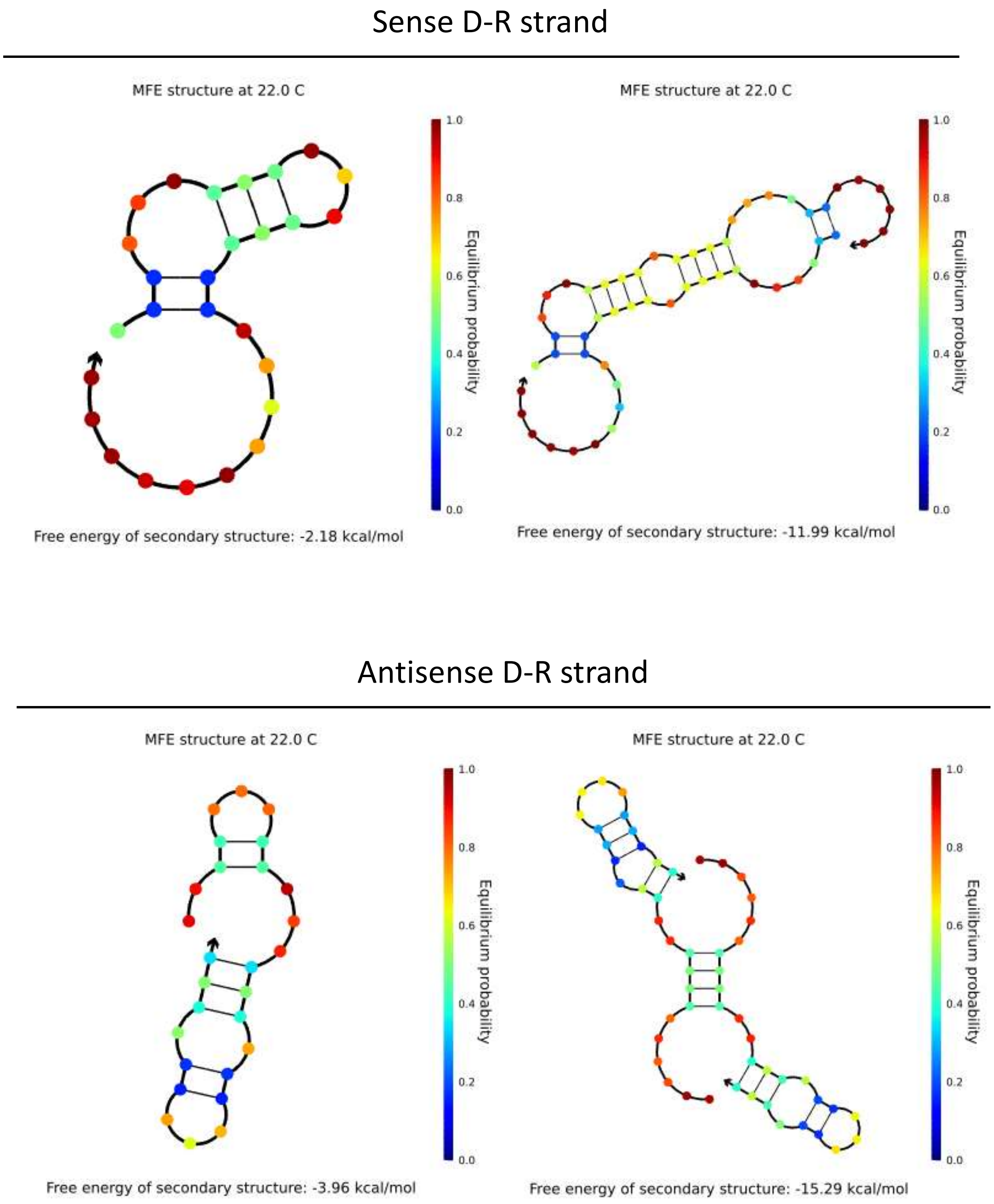

Figure S2. Thermodynamic intramolecular folding and intermolecular strand interaction analysis of Sense and Antisense A D-R strands (GFP) sequences performed with NUPACK (www.nupack.org) with RNA settings at 22° C and 1 µM concentration of strands. Only the corresponding RNA sequence was used as input considering that the D-R strand DNA regions would be hybridized to the DNA dendron arms.

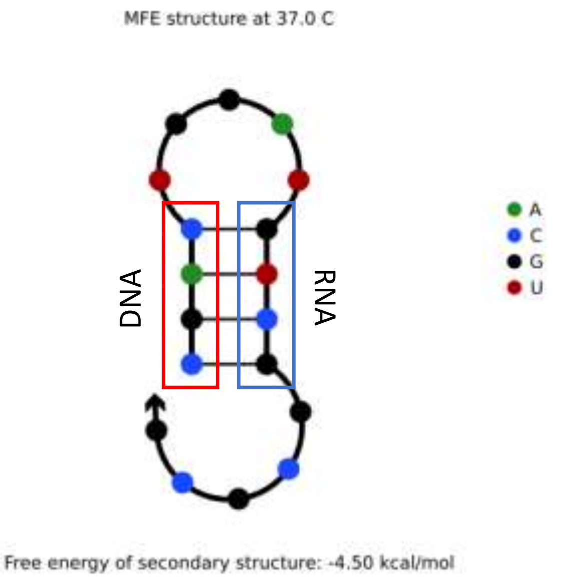

Sense D-R strand version I or II (GFP). Only the complementary region to DNA dendron arm is analyzed.

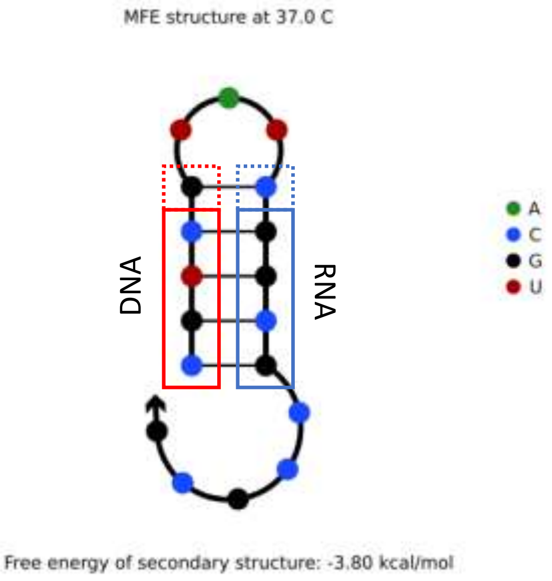

Antisense D-R strand version I or II (GFP). Only the complementary region to DNA dendron arm is analyzed.

Figure S3. Thermodynamic intramolecular folding analysis of the sequence regions of the Sense and Antisense D-R strands versions I and II (GFP) which are complementary to the DNA Dendron arms. Analysis performed with NUPACK (www.nupack.org) with RNA settings at 37° C and 1 µM concentration of strands. The boxes highlight the regions where there is RNA-DNA hybrid double strand formation, which can be recognized by RNAse H in the *in vitro* cleavage assays. A minimum of four RNA nucleotides are always hybridized to a complementary DNA sequence. In the case of the Antisense D-R strand version II there is an additional RNA nucleotide (forming a 5 nt stretch highlighted by the dashed box) forming a 5 bp RNA-DNA hybrid.

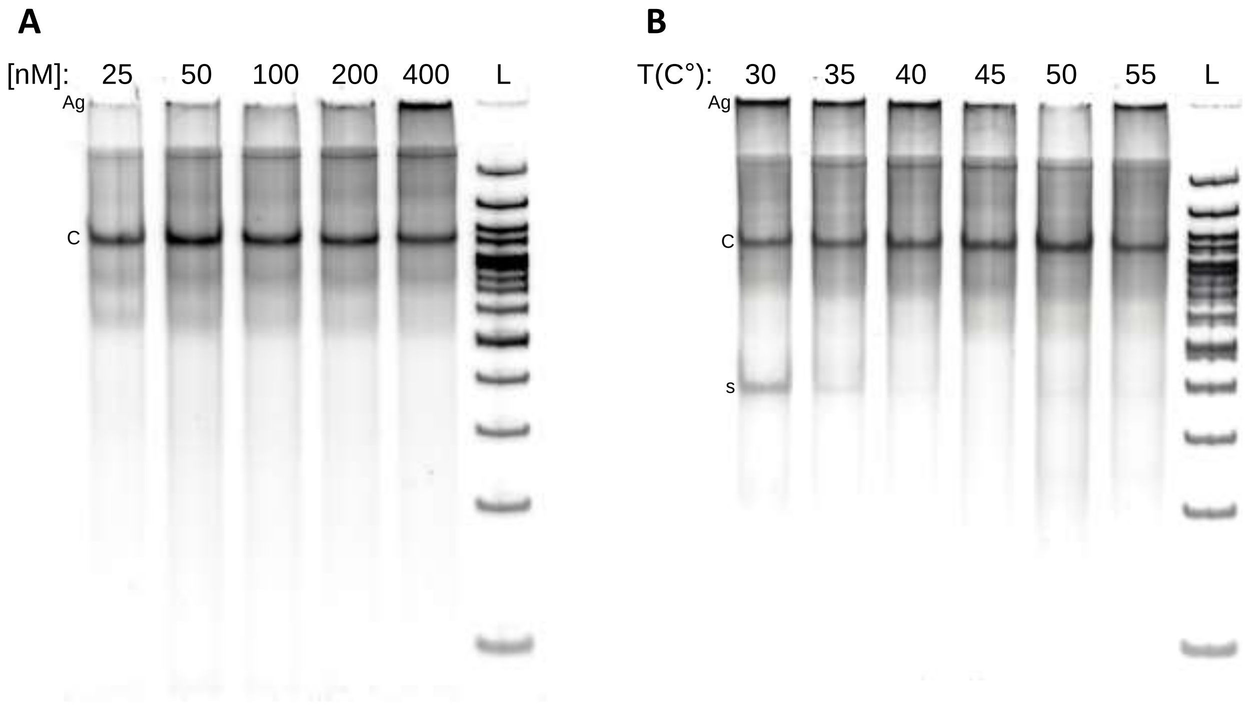

Figure S4. Polyacrylamide gel electrophoresis analysis of the second step in the CsiRNA assembly process. Branch S and Branch AS structures (formed by the Trebler A_S and Trebler A_AS with the respective sense and antisense D-R strands (GFP)) were left to hybridize at different annealing **A)** concentrations and **B)** temperatures, for formation of the closed caged structure - CsiRNA.

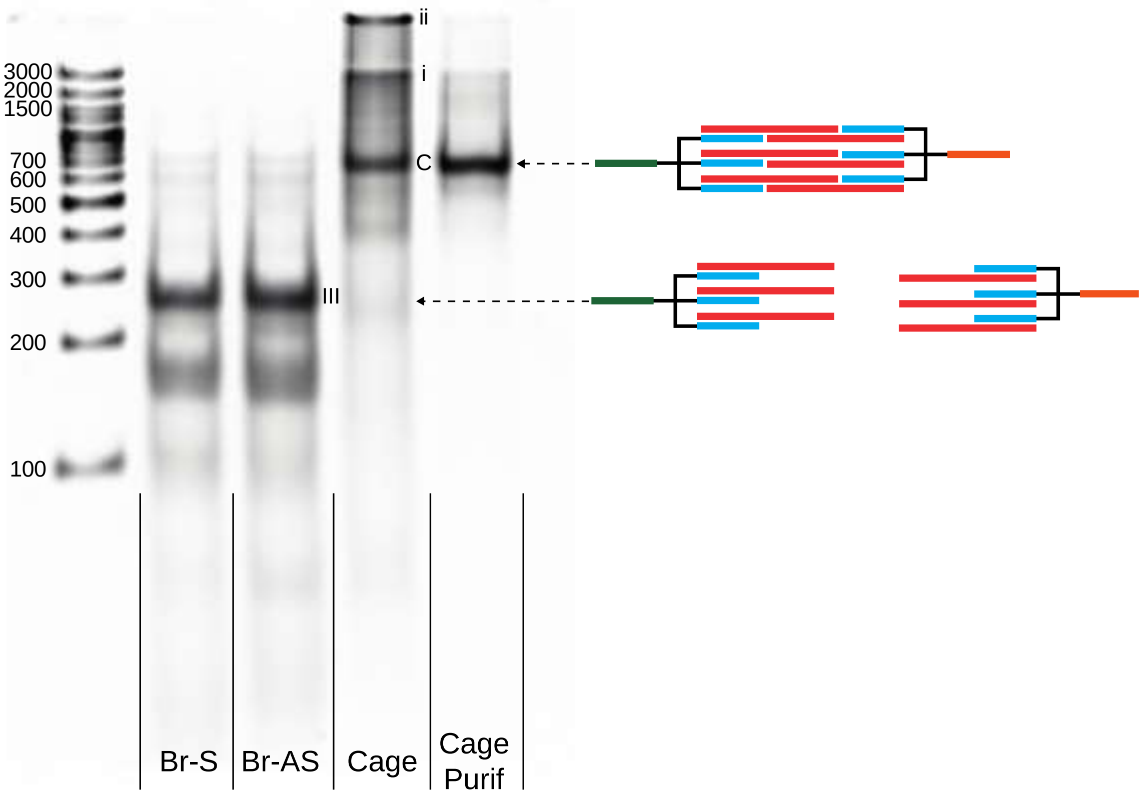

Figure S5. Polyacrylamide gel electrophoresis analysis of the H-CsiRNA assembly process and purification, with the RNAi sequences corresponding to anti-GFP. Annealing between Branch S (Br-S) and Branch AS (Br-AS) results in the preferential formation of a higher-molecular-weight band (band denoted by the letter "C") attributed to the closed Cage structure. Some additional higher molecular weight bands, in a lower proportion, are seen that can correspond to higher number (>2) of Branch S and AS units forming an interlocked structure (e.g., band "I"). Also, larger concatemers of several Branch (e.g., band "ii") can be observed that are stuck in the well. The cage band ("C") can be isolated and gel purified ("Cage Purif" lane) with no disruption of its migration in the gel thus, with no apparent alteration of the primary structure.

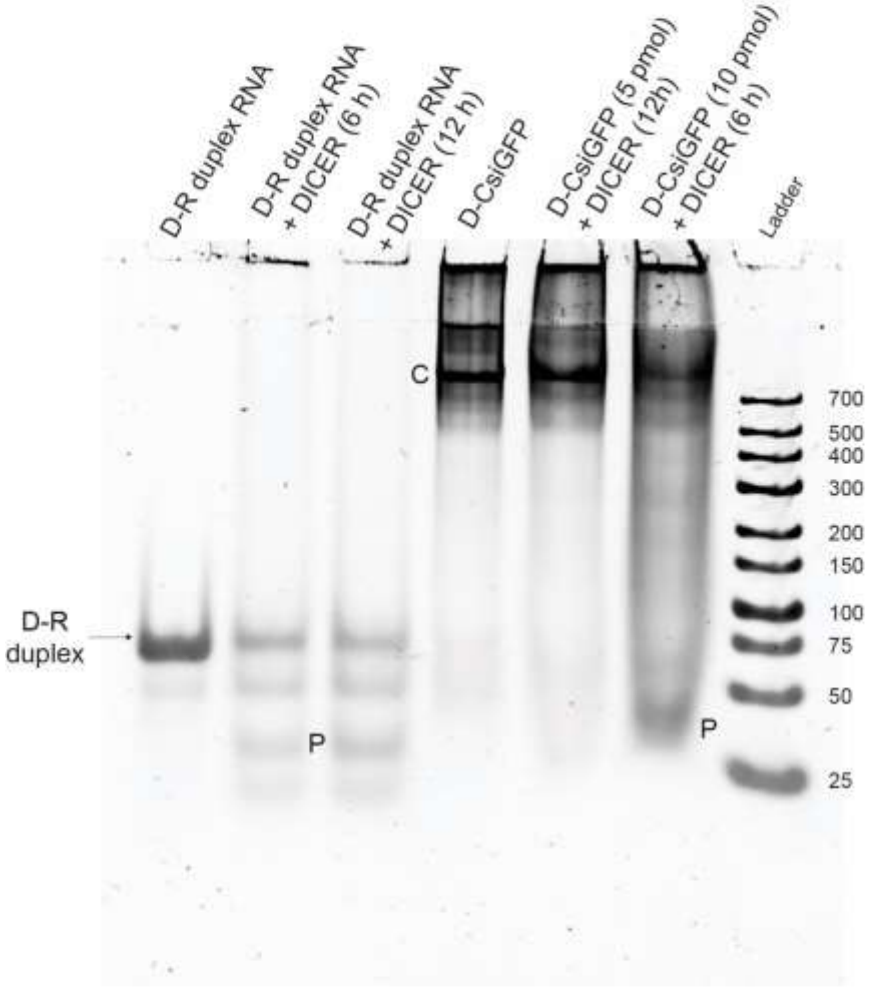

Figure S6. Second example of an in vitro cleavage assay of D-CsiRNAs by Dicer. **A)** Native PAGE stained by SybrGOLD showing the D-CsiRNA structure incubated with recombinant Dicer enzyme. The core region of the CsiRNA containing the hybridized D-R strands S and AS was used as a cleavage control and is indicated by the arrow (some left over, not completely hybridized, bands are visible but do not influence the enzymatic step). The cage band is indicated by "C" and a low-molecular-weight band product is indicated by "P". Two different amounts of D-CsiRNA were loaded with different DICER incubation times, however this in vitro process seems of quite low efficiency and variable (which is already observable by the incomplete digestion of the D-R duplex band used as control)
